# Beyond anxiety and face: strategic silence as instrumental disengagement in a Chinese graduate EAP Reading classroom

**DOI:** 10.3389/fpsyg.2026.1894201

**Published:** 2026-08-27

**Authors:** Feiyang Tian

**Affiliations:** Foreign Languages Department, Beihang University, Beijing, China

**Keywords:** classroom silence, EAP (English for academic purposes), instrumental motivation, perceived course value, self-efficacy, strategic disengagement

## Abstract

**Introduction:**

Silence in East Asian English classrooms has traditionally been attributed to “anxiety” and “face.” However, these factors may not sufficiently explain silence in our graduate EAP reading classroom where the course was deliberately designed as a four-week intensive module to teach essential academic reading skills while minimizing the burden on students. Yet despite this careful design, silence remained pervasive. This study is to test the hypothesis that silence in this obligatory, condensed EAP situation is a calculated strategy shaped by students’ perceived value of the course. Any academic requirement unrelated to their core research direction is perceived as unworthy of substantial time or energy investment.

**Method:**

A mixed-method approach was adopted. Survey analysis quantified and evaluated the relative predictive power of Perceived Course Value, Reading Anxiety, and Self-Efficacy on Silence Propensity. Focused group discussion investigated how students themselves rationalize their silence.

**Results:**

Reading Anxiety and Perceived Course Value scored a mean of 3.47 (SD = 0.69), 2.80 (SD = 1.05). Both exhibited a positive correlation with Silence Propensity (Perceived Course Value: *r* = 0.507, *p* < 0.01; Reading Anxiety *r* = 0.229, *p* < 0.01). Correlations among other variable pairs failed to reach statistical significance. The interaction term between Course Value Perception and Self-Efficacy is not significant in predicting silence tendency (*B* = − 0.0300, *t* = − 0.3202, *p* = 0.7494). Together, the three predictors—Perceived Course Value, Reading Anxiety, and Self-Efficacy—explained 31.6% of the variance in Silence Propensity (*R*^2^ = 0.316). Qualitative data demonstrated multifaceted reasons for the low ratings of the course.

**Discussion:**

Perceived Course Value was the strongest predictor of Silence Propensity. The non-significant correlation between Self-Efficacy and Silence Propensity is further addressed in the Discussion. The quantitative and qualitative evidence taken together presents a comprehensive depiction of EAP classroom silence, highlighting “rational disengagement” as a key mechanism and providing an alternative interpretation to the traditional diagnostic of “cultural shyness” in this specific EAP context.

## 1 Introduction

Against the backdrop of the declining prominence of English education in China (Fan, 2023; Yuan, 2021; Zhao, 2016)—evidenced by the dissolution of English departments across numerous universities and the attempt by legislators to limit English study time and reduce its weight on China’s university-entrance exams (The Economist, 2024)—the perceived value and engagement with English courses have experienced a notable shift compared to previous eras, although both instructors and students acknowledge the indispensable function of English as a lingua franca for informal and academic global communication.

Such a recalibration at the systemic level yields concrete implications for how students interpret the institutional signals embedded in curriculum design. In response to this changing educational landscape, we have developed a concise four-week English for Academic Purposes (EAP) reading course. This curriculum is designed to impart essential academic reading skills while minimizing interference with students’ research priorities. A key underlying assumption for faculty and administrators is that the compressed format will facilitate course completion before learner engagement diminishes, thus mitigating the gradual disengagement and attrition typically seen in longer-duration programs (Li, 2022; West, 2025). Yet when an EAP course is administratively streamlined in this way, students could interpret the format as an institutional recognition that the course is secondary rather than as a sign of efficiency.

Instructor observational records during the four-week course revealed low voluntary participation: across eight sessions, an average of only four to six students (out of about 125) answered open-ended questions without being called upon; wait times after teacher prompts frequently exceeded 10 seconds; and the majority of verbal contributions came from the same small subset of students. Instead of sporadic or anxiety-driven hesitation, these trends point to a persistent culture of instrumental disengagement. This phenomenon presents a critical puzzle. Why does a design aimed at optimizing learning efficiency seem to promote strategic disengagement? While anxiety and “face” concerns have been well documented as key predictors of silence in general EFL classrooms (e.g., Hanh, 2020; Harumi, 2024; King and Harumi, 2020; Zhuang, 2023). The majority of previous research has concentrated on undergraduate or younger students. It remains unclear whether these frameworks apply equally to graduate students in EAP contexts, where learners have different academic priorities and motivational orientations. This study therefore explores alternative explanations for silence in this under-studied population.

As the silence observed resembles not nervous avoidance, but rather the quiet of strategic minimalism, I therefore hypothesize that in this obligatory, highly condensed EAP situation, silence is not predominantly a manifestation of emotional or cultural barriers, but rather a calculated strategy shaped by students’ perceived value of the course. To test this hypothesis, this study adopts a dual-perspective approach. An etic (researcher-driven) analysis will quantify and evaluate the relative predictive power of Perceived Course Value, Reading Anxiety, and Self-Efficacy on Silence Propensity. Additionally, an emic (participant-driven) analysis will investigate how students themselves rationalize their silence, offering insider perspectives on their classroom experiences. By synthesizing these two perspectives, this study seeks to supplement the widely accepted affective-cultural paradigm, through constructing a more situationally grounded interpretation of silence in this distinct EAP environment. Specifically, two research questions guide this inquiry

1. How do Perceived Course Value, Reading Anxiety, and Self-Efficacy relate to Silence Propensity, both individually and collectively, in this EAP context?
2. How do graduate students themselves rationalize their silence in this specific EAP setting?

## 2 Literature review

### 2.1 The established paradigm: affect and culture

Students’ silence in language classrooms has been extensively studied as a multifaceted phenomenon. A substantial body of research has approached silence from a primarily affective perspective, emphasizing the importance of language anxiety—the fear of being judged negatively and communication anxiety—as a major barrier to oral participation (King and Harumi, 2020). In addition to this line of inquiry, a sociocultural strand of research has highlighted the impact of cultural norms prevalent in East Asian classrooms, such as collectivism, high power distance, and the cultural value of modesty and restraint (Zhuang, 2023). These norms may encourage students to participate cautiously and conservatively and to be reluctant to voice unique opinions (Hanh, 2020; Harumi, 2024; Liu, 2005, 2006). In China, for instance, the high-power-distance culture prioritizes collectivism and collective harmony over individual expression. This prompts students to engage in classroom discussions cautiously and conservatively, frequently opting to conform to mainstream views instead of expressing distinctive viewpoints (Zhuang, 2023). Moreover, cultural traditions that value restraint and modesty have nurtured inherent shyness in Asian students, making them reluctant to express themselves (Liu, 2005, 2006; Pham et al., 2023). In addition to this, systemic issues such as inadequate opportunities for meaningful language input and output, together with the predominant emphasis on written skills in university admission examinations, result in numerous learners being ill-prepared and lacking confidence in oral communication (Han, 2016; Hanh, 2020). Together, these factors create a complex web of barriers that inhibit active participation.

While these overlapping factors collectively contribute to classroom silence, the dominant explanatory framework for silence in East Asian EFL contexts remains bifocal. First, extensive research has documented a definitive link between foreign language anxiety and communicative withdrawal (Maher and King, 2022, 2023; Shachter and Haswell, 2022). This line of inquiry has been further corroborated by studies on Chinese learners, which consistently identify insufficient oral proficiency as a primary reason for classroom silence (Peng, 2024; Su and Chu, 2024; Wei and Cao, 2024; Yang and Lin, 2024; Zhuang, 2023)—a linguistic deficit that undermines self-confidence and intensifies avoidance behavior. Extending this affective viewpoint, recent work has also highlighted the role of social anxiety, a psychological condition characterized by fear of negative evaluation and evasiveness in performance-related scenarios, as an additional barrier to verbal engagement (Liu et al., 2026). Notably, social anxiety is closely intertwined with the second core focus of this discussion: the cultural concept of “face.”

In collectivist societies like China, where cultural norms emphasize “face,” conformity, and respect for hierarchy, evaluative concerns among individuals are significantly amplified. This cultural context not only cultivates social anxiety among university students but also renders “face” a crucial element shaping their classroom behavior. Drawing on Hofstede’s cultural dimensions and Confucian pedagogical principles, numerous researchers have recognized “face” as a fundamental social heuristic that guides student interactions (Ahmed, 2024; Bao, 2014; Langen and Stamov Roßnagel, 2023; Zhu and O’Sullivan, 2022). The passive and reticent performance observed among Chinese students is often ascribed to the deeply rooted Confucian values (Chen, 2020). Both low- and high-achieving students may remain silent due to face-related concerns: the former to avoid shame, embarrassment, and exposure of inadequacy, the latter to prevent perceptions of boastfulness, arrogance or immodesty among peers (Chen, 2020; Zhao, 2018). In addition, the Chinese classroom is typically characterized by high formality and hierarchical structure, with the teacher occupying a position of authority, and with an emphasis on rote learning and passive information reception. This power imbalance discourages questioning or critical engagement, fostering an environment where students have limited opportunities for expression and are hesitant to dispute or communicate with instructors (Wang, 2019). To summarize, silence in Chinese EFL classrooms “can be understood as a culturally influenced response to deeply rooted norms and social expectations” (Alhetah and Alharbi, 2026:3).

### 2.2 The critical gap: motivation, context, and learner agency

This affective-cultural paradigm provides a compelling framework for understanding student silence; yet it neglects several essential elements. First, this paradigm overlooks the role of instrumental motivation, which drives learners to prioritize practical outcomes—such as passing exams, meeting academic requirements, or securing employment—over the intrinsic value of language acquisition (Wang, 2019). When students are motivated primarily by external rewards rather than genuine interest, silence may become a conscious decision to withdraw from participation, rather than a reaction to cultural or affective pressures (Pham et al., 2023).

A second, closely related factor is self-efficacy, a key construct in Bandura’s social cognitive theory (Bandura, 1986). Self-efficacy refers to individuals’ belief in their capacity to succeed in specific tasks; those with low self-efficacy in academic English are more likely to perceive participation as a futile or high-cost endeavor, and thus withdraw from classroom interactions (Yue et al., 2022). While instrumental motivation concerns what students hope to gain by staying silent, self-efficacy concerns whether students believe they are capable of contributing meaningfully.

Third, learner agency—the ability of students to act as logical decision-makers who weigh costs and benefits in their learning environment—is often overlooked by the affective-cultural paradigm, which treats students as passive recipients of cultural scripts or emotional states. According to this perspective, silence is not merely a reaction triggered by anxiety or cultural norms; it can also reflect a purposeful tactic based on students’ evaluation of what is worth their time and effort. Therefore, silence is not only associated with passivity; it should be understood as a process of languaging within specific interactions and cultural contexts (Alhetah and Alharbi, 2026). Taken together, these three concepts—instrumental motivation, self-efficacy, and learner agency—operate at different levels but converge to shape a common behavioral outcome: strategic disengagement from classroom participation.

In intensive EAP settings, students with low self-efficacy in academic English may regard the cognitive and time investments required for participation as unwarranted, prompting them to withdraw from classroom interactions. This cost–benefit reasoning suggests a more comprehensive theoretical perspective: students can be viewed as rational agents who actively navigate their learning environments by balancing the perceived value of participation against its costs, as opposed to being passive responders to cultural norms or emotional states. This study therefore characterizes students as rational agents who navigate institutional constraints such as course requirements and grading systems to strategically allocate their cognitive resources. This reframing challenges the long-held assumption that silence equals disengagement, instead positioning it as a communicative decision influenced by learners’ perceptions of the classroom context and cultural norms. From this perspective, inactive classroom participation is not merely a result of anxiety or cultural influence but a purposeful, learner-driven act of meaning-making, a deliberate, calculated choice of students weighing the potential costs and benefits of engagement against competing academic demands.

### 2.3 Proposed framework: the strategic disengagement model

Synthesizing the critiques above, this study proposes a Strategic Disengagement Model to understand classroom silence in mandatory, short-format EAP courses. This model is informed by two established motivational frameworks: Expectancy-Value Theory (EVT) and Self-Determination Theory (SDT). From an EVT perspective (Eccles and Wigfield, 2002), students engage in a cost–benefit analysis when deciding whether to participate. Two key components of EVT are central here: subjective task value and cost. Students are hypothesized to weigh the perceived expense of active engagement against the course’s benefit. When the value is low and the cost is high, silence becomes a rational outcome. This echoes the EVT logic that behavior depends on both subjective task value and expectations for success. Meanwhile, SDT (Deci and Ryan, 2012) provides a lens to understand the quality of motivation driving this calculation. The model hypothesizes that students in this context are characterized by controlled motivation—their engagement is driven by external contingencies (grades, credits) rather than intrinsic desire or recognized worth. The survey items measuring Perceived Course Value (e.g., “my main goal is to earn the credits”) capture this instrumental, externally-regulated orientation. Thus, silence is a behavior predicted by low-quality motivation that SDT would describe as non-autonomous, rather than just an EVT-driven calculation.

The Strategic Disengagement Model posits that students, as rational agents, engage in a cost–benefit analysis when deciding whether to participate. Three sets of variables are hypothesized to shape this calculation. First, Perceived Course Value operationalizes students’ response to institutional signals and their motivational orientation. A high score on the four-item scale (e.g., “my main goal is to earn the credits”; “if I had a choice, I would not take this course”) implies strong instrumental motivation, reflecting a “box-ticking” mindset. This scale is posited to be the primary driver of strategic disengagement: when students perceive the course as a low-stakes formality, silence becomes a rational choice. Second, Self-Efficacy reflects students’ confidence in their ability to perform EAP reading tasks. This variable may moderate the relationship between Perceived Course Value and silence, i.e., even students with low course-value-perception may still be less likely to disengage entirely if they have a high self-efficacy. Third, Reading Anxiety represents the affective dimension emphasized in the mainstream literature. While previous studies often treat anxiety as a primary cause of silence, this model assumes that in the specific context of a short, mandatory EAP course, anxiety plays a secondary role—it may intensify silence, but it is not the primary cause once Perceived Course Value is accounted for.

Notably, the construct of “face” which is central to the affective-cultural paradigm is not included in our survey. This was a deliberate choice based on pilot observations in which “face” was perceived as negligible among these adult graduate learners. They were more concerned with whether a question was intellectually enough to engage with than about peer judgment over a wrong answer. Thus, while we acknowledge the theoretical importance of “face” in the broader literature, our empirical focus here is on the variables that emerged as salient in this specific context. In sum, the Strategic Disengagement Model postulates a set of relationships among Perceived Course Value, Reading Anxiety, Self-Efficacy, and Silence Propensity, with each variable occupying a distinct theoretical role. The following section describes the mixed-methods design used to test this model.

## 3 Materials and methods

An explanatory mixed-methods approach was adopted to collect quantitative as well as qualitative data. Quantitative data were gathered via a structured questionnaire administered through Wen Juan Wang (Questionnaire Network), a professional online survey and voting platform designed for questionnaire creation, distribution, and data analysis. Data collection was conducted in a controlled classroom environment to guarantee a high response rate and reduce response bias. 10 min before the end of a regular class, students were instructed to scan a QR code leading to the online questionnaire, with a 10-min period designated for completion. Upon completion, the instructor accessed and examined the dataset directly via the platform’s built-in data management system, which enabled initial data cleaning and descriptive statistical analysis.

A random sample of nine students was chosen for semi-structured follow-up interviews to contextualize and elaborate on the quantitative findings. This qualitative phase sought to investigate the fundamental mechanisms and intricate relationships between students’ academic performance and essential psychological constructs, such as intrinsic and extrinsic motivation, foreign language reading anxiety, and attitudinal orientations toward the curriculum. The interview protocol was created based on emergent themes from the quantitative data, ensuring alignment between the two stages of the study and facilitating a holistic comprehension of the research phenomenon.

### 3.1 Context and participants

The study was embedded within a 4-week, 8-session “Academic Reading Strategies” module offered to first-year Master’s students at Beihang University. The complete cohort, consisting of 120 students in four classes from various disciplinary backgrounds, constituted the quantitative sample. All participants were enrolled in natural science disciplines, including Power Engineering, Materials Science, Physics, Aerospace Propulsion, Energy and Power Engineering, Mechanical Engineering, and Transportation Engineering. Most students successfully completed the College English Test Band 6 (CET-6), and some have also achieved qualifying scores in TOEFL and IELTS, indicating intermediate to advanced English language competency. The questionnaire was presented during the final session of the fourth week of instruction, which was deliberately selected to enable students to contemplate their whole experiences about the course design, instructional techniques, and learning results over the full duration of the program.

### 3.2 Data collection instruments and procedure

The integrated Online Questionnaire consists of two sections: Part I solicits participants’ personal information, including their major and their proficiency in English. Part II incorporates two validated scales and two adapted scales: Scales 1 and 2 (about Reading Anxiety and Self-Efficacy) are validated instruments extensively used in previous studies which reported acceptable levels of internal consistency (e.g., Chemers et al., 2001; Dang, 2024; Hemade et al., 2025; van Zyl et al., 2022). Scales 3 and 4—Perceived Course Value and Silence Propensity—were developed specifically for this study to capture constructs not adequately addressed by existing instruments in the context of graduate EAP classrooms. Item generation was based on observations made in the classroom and casual conversations with graduate students regarding their opinions of EAP courses and the reasons behind their non-participation. Two applied linguists then evaluated an initial set of items to determine their content validity, clarity, and relevance to the target constructs. Items were revised or eliminated to improve precision based on their input.

A pilot study was then conducted with 27 graduate students from a comparable EAP course. Item analysis was performed by examining corrected item-total correlations and the effect of item deletion on Cronbach’s *α*. Items with low corrected item-total correlations (<0.30) were removed. The final version of the Perceived Course Value scale comprised four items: (1) “My main goal in this course is simply to earn the credits; I do not care how much I actually learn”; (2) “If I had a choice, I would definitely not take this course”; (3) “Compared to my major courses, this one has very low priority in my mind,” and (4) “I feel this course is just a formality set up by the university, with little real value for graduate students like us.” The Silence Propensity scale comprised three items: (1) “In this class, I usually do not speak up unless the teacher calls on me”; (2) “Even when I have something to say, I tend to remain silent,” and (3) “I believe listening carefully in class is enough; there is no need to participate in discussions.” All questions were presented in the form of a Likert scale, ranging from 1 (strongly disagree) to 5 (strongly agree). Cronbach’s *α* coefficients for the four subscales in the pilot study were 0.885, 0.814, 0.930, and 0.852, respectively, indicating acceptable internal consistency. Only slight wording modifications were made before formal administration.

Following the completion of the four-week teaching, two 30-min focus group interviews were conducted outside of regular class hours. Random sampling was employed to recruit two participant groups: one consisting of 4 students and the other of 5. Participants were initially identified through random selection from the survey respondents and then invited voluntarily. This approach may have introduced a self-selection bias, as only those willing to share their experiences participated. However, I observed that the final sample of interviewees still included a variety of viewpoints, including students who expressed instrumental disengagement, described silence as a habit, and attributed it to comprehension issues. This suggests that the data captured meaningful variation. The interviews followed a semi-structured approach aimed at obtaining comprehensive, contextually rich data across four thematic domains: (1) Participants’ overall impressions and evaluative feedback concerning the course design, instructional methods, and teacher facilitation strategies; (2) Antecedents of classroom silence, including individual, interpersonal, and contextual factors; (3) Assessment of the perceived value of in-class discussions versus individual independent work activities, focusing on cognitive, affective, and behavioral results; (4) Adaptive coping mechanisms and disengagement techniques employed by students to fulfill minimal course requirements while minimizing active participation.

In addition to the questionnaire and interviews, the instructor also recorded observational field notes during the four-week program. These notes documented intricate classroom dynamics that quantitative measures could not. Focus was directed toward recurring patterns—specifically, the indicators of student disengagement (e.g., looking down, checking phones, packing up early) or, on rare occasions, engagement (e.g., leaning forward, spontaneous note-sharing). The observational data offered ecological validity and facilitated the connection between students’ survey responses and real classroom occurrences.

### 3.3 Data analysis

Quantitative data were examined utilizing SPSS. Reliability was evaluated using Cronbach’s *α*. Descriptive statistics and Pearson correlations were calculated for all variables. A multiple linear regression analysis was conducted using Silence Propensity as the dependent variable and Reading Anxiety, Self-Efficacy, and Perceived Course Value as independent factors. One-way ANOVA was employed to compare English proficiency groups, and moderation analysis was conducted using the PROCESS macro (Model 1) to examine the moderating effect of Self-Efficacy.

Following an inductive approach, qualitative data (interview transcriptions) were analyzed using reflexive thematic analysis (Braun and Clarke, 2012), with themes generated from the data rather than imposed *a priori*. The qualitative findings were employed to contextualize and enhance the interpretation of the quantitative data, especially in explaining the underlying mechanisms of students’ strategic disengagement.

## 4 Findings

### 4.1 Quantitative results

A total of 120 participants answered the questionnaire, and 115 valid responses were collected. Quantitative data were analyzed in the following steps. First, Harman’s single-factor test was used to assess common method bias. Second, internal consistency reliability was examined using Cronbach’s *α* for all four scales, followed by exploratory factor analysis (EFA) to evaluate the structural validity of the two newly developed scales—Perceived Course Value and Silence Propensity. Third, descriptive statistics and Pearson correlations were calculated for all key variables, ‌succeeded by a multiple linear regression analysis. Fourth, one-way ANOVA was used to examine differences across English proficiency groups. Finally, moderation analysis using the PROCESS macro (Model 1) was performed to test whether Self-Efficacy moderated the relationship between Perceived Course Value and Silence Propensity.

#### 4.1.1 Preliminary analyses

Harman’s single-factor test demonstrated that the first unrotated factor accounted for 28.86% of the total variance, which was below the 40% threshold. Therefore, common method bias was not a serious concern. Reliability analysis revealed acceptable internal consistency for all scales (Cronbach’s α: Reading Anxiety = 0.64, Self-Efficacy = 0.70, Perceived Course Value = 0.88, Silence Propensity = 0.84). For the two newly developed scales—Perceived Course Value and Silence Propensity—exploratory factor analysis (EFA) was conducted to assess structural validity. For Perceived Course Value (four items), KMO = 0.802, Bartlett’s test was significant (*p* < 0.001), and a single factor explained 73.08% of the variance. For Silence Propensity (three items), KMO = 0.711, Bartlett’s test was significant (*p* < 0.001), and a single factor explained 75.64% of the variance. These results support the unidimensionality of both scales.

#### 4.1.2 Descriptive statistics and correlation

The descriptive statistics pertaining to each variable are summarized in Table 1. With a mean score of 3.47 (SD = 0.69), Reading Anxiety was found to be at a moderately high level (M = 3.47, above the midpoint of the 5-point scale). Self-Efficacy, with a mean of 3.55 (SD = 0.73), also reached a moderately high level. Perceived Course Value scored a mean of 2.80 (SD = 1.05). Silence Propensity had a mean of 3.28 (SD = 0.99), also at a moderately high level.

**Table 1:** Descriptive statistics of key variables (*N* = 115).

Variable	Minimum	Maximum	Mean	SD
Reading anxiety	1.40	5.00	3.47	0.69
Self-efficacy	2.00	5.00	3.55	0.73
Perceived course value	1.00	5.00	2.80	1.05
Silence propensity	1.00	5.00	3.28	0.99

Pearson correlation analysis was conducted to examine the relationships among the key variables, with findings displayed in Table 2. Reading Anxiety exhibited a significant negative correlation with Self-Efficacy (*r* = −0.336, *p* < 0.01) and a positive correlation with Silence Propensity (*r* = −0.229, *p* < 0.01), suggesting that higher levels of reading anxiety are associated with lower self-efficacy and an increased tendency toward silence. Perceived Course Value exhibited a significant positive correlation with Silence Propensity (*r* = 0.507, *p* < 0.01). Correlations among other variable pairs failed to reach statistical significance. Specifically, Reading Anxiety showed no significant correlation with Perceived Course Value (*r* = 0 0.021), Self-Efficacy was not significantly correlated with Perceived Course Value (*r* = 0.109), and Self-Efficacy also had no significant correlation with Silence Propensity (*r* = 0.163).

**Table 2:** Correlation table among variables (*N* = 115).

Variable	Reading anxiety	Self-efficacy	Course value perception	Silence propensity
Reading anxiety	1			
Self-efficacy	−0.336	1		
Course value perception	0.021	−0.109	1	
Silence propensity	0.229	−0.163	0.507	1

#### 4.1.3 Regression analysis

To examine the predictive effects of the three variables on Silence Propensity, a multiple linear regression analysis was conducted with Silence Propensity as the dependent variable and Reading Anxiety, Self-Efficacy, and Perceived Course Value as independent variables (Table 3).

**Table 3:** Multiple linear regression results for silence propensity (*N* = 115).

Variable	*B*	SE	*β*	*t*	*p*
(Constant)	0.925	0.731		1.266	0.208
Reading Anxiety	0.330	0.120	0.229	2.747	0.007
Self-Efficacy	−0.041	0.114	−0.030	−0.360	0.719
Perceived Course Value	0.481	0.075	0.509	6.429	< 0.001

The regression model demonstrated statistical significance (*F* = 17.059, *p* < 0.001), suggesting that at least one of the three predictors significantly predicted Silence Propensity. The model exhibited a *R*^2^ of 0.316 and an adjusted *R*^2^ of 0.297, indicating that the three independent variables collectively accounted for 31.6% of the variance in Silence Propensity. The unstandardized coefficients (B) for the three predictors were 0.330 (Reading Anxiety), −0.041 (Self-Efficacy), and 0.481 (Perceived Course Value), with a constant of 0.925. Perceived Course Value was the most significant predictor of Silence Propensity (*β* = 0.509, *t* = 6.429, *p* < 0.001). Reading Anxiety exhibited a positive predictive effect (*β* = 0.229, *t* = 2.747, *p* = 0.007). Self-Efficacy did not substantially predict Silence Propensity (*β* = −0.030, *t* = −0.360, *p* = 0.719). Collinearity diagnostics showed that all three independent variables had variance inflation factors (VIF) ranging from 1.016 to 1.145, all well below 10, and tolerance values ranging from 0.873 to 0.984, all above 0.1. These results mean that multicollinearity was not a serious concern, and the regression model estimates are reliable.

#### 4.1.4 Additional analysis

One-way ANOVA revealed no significant differences across English proficiency groups in Perceived Course Value or Silence Propensity. Reading Anxiety differed significantly, with the IELTS group reporting lower anxiety than CET6 groups (*p* = 0.011). Similarly, significant differences were found across groups in terms of Self-Efficacy (*F* = 9.537, *p* < 0.001). Overall, higher English proficiency was associated with higher self-efficacy. In addition, moderation analysis was conducted to examine whether Self-Efficacy moderated the relationship between Perceived Course Value and Silence Propensity. We hypothesized that when higher Course Value Perception is associated with higher Silence Propensity, Self-Efficacy would significantly weaken the predictive power of Perceived Course Value on Silence Propensity. Contrary to this hypothesis, results showed that Course Value Perception has a significant positive predictive effect on silence tendency (*B* = 0.4778, *t* = 5.7860, *p* < 0.001). The interaction term between Course Value Perception and Self-Efficacy is not significant in predicting silence tendency (*B* = −0.0300, *t* =  −0.3202, *p* = 0.7494). Self-Efficacy did not influence the relationship between Perceived Course Value and Silence Propensity.

### 4.2 Qualitative themes

The interview data reported below provide contextual explanations for the low course valuation observed quantitatively, offering insights into the specific reasons that underpin students’ instrumental disengagement. A sample of 9 students was randomly selected and subsequently divided into two focus groups, where semi-structured interviews were conducted to elicit their perspectives on the course content and their perceptions of classroom silence. Each interview session lasted about 30 min, with detailed field notes documented in a research diary and verbatim transcription completed immediately post-interview. Thematic analysis of the interview transcripts revealed three overarching themes regarding students’ perceptions of the EAP course and their silence behavior.

#### 4.2.1 Perceptions of course value

The results were not entirely consistent with our initial hypothesis that the course would be perceived as marginal, credit-bearing requirement with minimal intrinsic value. Four of the nine participants indicated tangible academic improvements resulting from the four-lecture module, emphasizing the practical applicability of the knowledge gained for their future studies.

S3: 这门课程让我清晰的理解了学术英语写作的一般框架，而这是我在平时不会主动研究的。

*This course provided me with a clear understanding of the general framework of academic English writing, which I would not have actively studied on my own.*

S7: 对，我现在对学术论文的结构有了更清晰的认识，会把课上学的东西，分析方法等用到科研任务中去。

*Yes, I now have a clearer understanding of the structure of academic papers and will apply what I've learned in class, such as analytical methods, to my research tasks.*

S4: 四周课时有点少，不能形成系统性的知识框架，对于科研阅读来说，只能给予一些理解论文的技巧，建议加长课时。

*The current four-week class arrangement is insufficient to establish a systematic knowledge framework. For scientific research reading, it can only provide some techniques for understanding papers. It is recommended to extend the class hours.*

S9: 我上这个课之前，听过去的同学说没用，做完作业，通过考试拿到学分就好了。但我觉得不是这样的，学到的东西对平时的专业文献阅读还是很有帮助的。

*Before taking this course, I heard from other classmates that it was useless—just finish the assignments and pass the exams to get the credits. But I don’t think it’s that way. The knowledge gained is very helpful for reading research articles.*

Student 4 stated that the four-week course duration was not enough for him to achieve the goal of reading and interpreting academic papers easily and effortlessly. Two other participants agreed with this view, saying that they felt they had not acquired substantive knowledge because the course ended too soon. However, in contrast to these positive reflections, five participants gave negative feedback, stating that they did not perceive the course as beneficial, and they would prefer to withdraw from the course if given the choice.

#### 4.2.2 Reasons for low perceived course value

Participants gave specific explanations for why the course remained low in their academic priorities, even though they acknowledged some learning gains. There were three sub-themes. First, one student noted that the course material did not match their real academic demands. Second, three students mentioned that the availability of AI tools further reduced the perceived necessity of formal EAP training. Third, the course increased a workload that was already too much.

S1: 但在我的研究方向不需要阅读很多英文文献，毕业要求也不需要写英文论文，所以可能学到的知识没有应用途径。

*But in my research direction, I don't need to read a lot of English literature, and there is no requirement to write an English thesis for graduation, so what I have learned from this course may not have a practical application.*

S2: 现在AI辅助读论文写论文都很方便，所以不是很想上，感觉对以后的生活没什么用，但是有些讲到的知识也还行，比如IMRD啥的，但是还是觉得这个课没必要，如果能不选我会选择不选。

*Nowadays, AI assisted reading and writing of papers are very convenient, so I don't really want to take the course. I feel that it is not very useful for my future life, but some of the knowledge covered is also okay, such as IMRD structure format. However, I still feel that this course is unnecessary. If I could choose not to take it, I would.*

S5: 虽然愿意去学习英语，但是老师给的科研压力又特别大，上英语课反而成为了一种负担。

*Although I’m willing to learn English, the research pressure imposed by teachers is particularly high, and attending English classes has become a burden.*

These testimonies indicate that students’ low perceptions of the course value resulted from a contextual evaluation that the course offered little return on investment to their immediate scholastic demands rather than from a rejection of English per se. Student 2 noted that AI tools offer a more convenient and accessible substitute for formal instruction, making the EAP course feel unnecessary. Two other participants echoed this view in their interviews: AI时代来临, 语言的隔阂越来越小 (The AI era has arrived, and language barriers are becoming increasingly smaller.); 现在都是AI辅助阅读, 理解文章是没问题的, 上不上英语课没必要(Nowadays, AI-assisted reading makes understanding easy, so English classes are not so necessary). In contrast to these who struggled with comprehension or relied on AI tools, student 8 expressed confidence in his ability to understand academic articles:感觉并没有很玄幻, 都是可以理解的 (the course content did not feel particularly mysterious—it was all understandable). For this participant, the course appeared to offer limited new learning, and his low perceived value stemmed not from difficulty or irrelevance, but from a perceived redundancy relative to his existing competence. Meanwhile, student 6 exhibited minimal responsiveness throughout the interview. When asked to explain his reticence, he said, 没什么感觉, 就是一门英语课 (I do not feel anything about it—it’s merely an English class.) This view may reflect the common perception among graduate students that English plays a peripheral role in academic evaluation, as their priority is scientific research rather than language skills. It also points to the fact that numerous students have been subjected to obligatory English education since elementary school, and extended exposure to required instruction has cultivated a sense of indifference. Therefore, disengagement—manifested as silence—has become a habitual behavior, evident not only in formal classroom environments but also in targeted research interviews aimed at assessing an English language course.

#### 4.2.3 Silence as habit or strategy

As regards the causes of their in-class silence, five respondents ascribed it to habitual behavior, while three others attributed it to a lack of self-confidence arising from perceived inadequacies in their English competence.

S4: 习惯于自己思考而不去表达, 代表我当时还没搞明白, 不知道说什么。

*I’m accustomed to thinking on my own without expressing myself, it means that I didn't understand at the time and didn't know what to say.*

S7: 不知道, 就是一种习惯。从小到大养成的习惯。上其他课一样。

*I don't know, it's just a habit developed from childhood to adulthood. I’m also silent in other classes.*

S5: 对, 就是一种习惯, 一直都这样。

*Yes, it's just a habit, and it's always been this way.*

S2: 英语不好, 对自己想法不自信, 不敢说出来, 也一直不太会用英语表达.

*My English is not good, I am not confident in my own ideas, I dare not speak out, and I am not very good at expressing myself in English.*

S1: 就是觉得安全，That means lower risk.

*I just feel safe in this way. That means lower risk.*

This habit-induced disengagement is echoed in previous research (e.g., Alhetah and Alharbi, 2026; Pham et al., 2023), suggesting that students’ previous educational experiences significantly influence their learning orientations during secondary and postsecondary education. Participants specifically reported that high school educational settings prioritized passive reception which persists in their university experience. This “habit” of silence does not necessarily contradict the Strategic Disengagement Model. In this case, habits may start off as deliberate strategies—students learn early on that silence is safer or more effective—and eventually become automatic. Thus, what seems like a passive habit could be the result of past calculated decisions or a rational adjustment to perceived costs and benefits.

In addition to habit-induced disengagement and insufficient confidence in English proficiency, Student 6 ascribed his classroom silence to a mismatch between his personal academic interests and the course reading materials. The generic, cross-disciplinary nature of the course texts failed to elicit his interest.

S6: 我个人是更注重理解一点，因为时间有限，只想细读感兴趣的文章,

专业对口的文章。

*I personally place more emphasis on understanding because time is limited, and*

I only want to read articles that interest me and are relevant to my field of study.

This underscores a challenge in curriculum design and the selection of suitable reading resources for heterogeneous learner groups. Due to the diversity of students’ academic majors, it is impossible to choose texts that satisfy every individual’s particular research interest. In addition, the participants identified excessive academic demands as an impediment to engaging with English language acquisition. They adopt a pragmatic, utilitarian approach to learning English, allocating time and cognitive resources to items deemed directly pertinent to their research requirements. Consequently, a disparity arose between curricular intention and student interpretation. The course was designed as an efficient, focused EAP sprint; but, students, preoccupied with their disciplinary obligations, regarded it as a secondary formality and exhibited strategic disengagement.

To summarize, thematic analysis of semi-structured interviews with nine randomly selected participants revealed three core insights concerning students’ perceptions of the course: (1) A notable proportion of students expressed a wish to improve their academic reading skills, yet concurrently recognized that the four-week course was insufficient to fulfill this objective. This indicates that significant enhancement in academic English is unattainable within 4 weeks, rendering trial-and-error involvement futile; (2) Students’ disengagement or disinterest in the course was generally ascribed to excessive academic burdens. Participants reported adopting a strategic approach for cognitive resource allocation, emphasizing tasks pertinent to their primary research objective and avoiding engagement with materials outside their immediate disciplinary scope; (3) Silence was identified as originating from two main sources. For some students, silence constituted an entrenched behavioral habit, reflecting a wider societal norm of passive engagement in the classroom. For others, silence stemmed from a lack of self-confidence in their English language skills.

Thus, different from the quantitative findings, which identified the dominant factor—low course value assessment as a significant predictor of students’ inclination toward silence—the qualitative results reveal a complex array of contributing factors ranging from the heavy burden of academic research, irrelevant reading materials, entrenched passive learning habits, to the perceived marginal status of English courses within the curriculum framework. The qualitative data enhances our understanding of classroom silence with greater depth and nuance: Although Perceived Course Value acts as the direct trigger of students’ silence, a confluence of underlying variables drives this devaluation.

### 4.3 Observational notes

Self-reported measures of silence propensity may not fully align with actual classroom behavior—a limitation known as the intention-behavior gap (Sheeran and Webb, 2016). To address this, structured classroom observations were conducted throughout the eight sessions. The observational findings reported below serve to triangulate the self-reported data.

Three recurrent patterns were identified. First, voluntary responses to open-ended questions were rare: across most sessions, only two to three students raised their hands or spoke without being nominated, with the same few individuals dominating what little verbal participation occurred. Second, the vast majority of students stayed silent when the instructor paused for remarks or questions, even among those who showed obvious comprehension through non-verbal cues like nodding, highlighting, or taking notes. This implies that the choice to keep silent was motivated more by reluctance than by a lack of competence. Third, a number of students casually remarked after class that they thought the EAP course was more of a “listening course” than a “speaking course,” suggesting that they did not see active oral involvement as part of their expected role. These observational data suggest that the self-reported silence propensity was broadly consistent with observed behavior, supporting the validity of the scale as a proxy for actual participation patterns in this specific context.

## 5 Discussion

This study set out to address two research questions. The first asked how Perceived Course Value, Reading Anxiety, and Self-Efficacy relate to Silence Propensity in this EAP context. The findings provide a clear answer, which has been detailed in the Results section. The second question explored how students themselves rationalize their silence. Interview data revealed four recurring justifications: the four-week format was seen as too short to warrant sustained effort; AI tools were viewed as rendering formal instruction redundant; the course was experienced as an additional burden on research workloads; and for a few proficient students, the content was simply unnecessary. Together, these arguments reinforce the logic of the Strategic Disengagement Model: silence is but a reasoned response to students’ assessment of the course’s value relative to its perceived costs.

As mentioned above, regression analysis identified Perceived Course Value as the strongest predictor of Silence Propensity, while Self-Efficacy has an insignificant predictive effect. This is in line with our hypothesis before: The low evaluation of the course value directly results in students’ lack of motivation to participate in the classroom activities. But the average score for Perceived Course Value was merely 2.80, which is below the midpoint of the 5-point scale, and qualitative feedback from certain students suggested they found the course beneficial, with some even stating that the four-week duration felt insufficient. These two observations are not contradictory. Instead of indicating ambivalence within students, a mean of 2.80 with a standard deviation of 1.05 indicates variation between students. This interpretation is supported by the qualitative data, which included both positive and negative accounts. More importantly, the seeming tension between low mean scores and the recognition of learning gains can be resolved by distinguishing between two types of course value. Our survey measures instrumental value (priority, credit-orientation), whereas qualitative comments addressed learning value (skill acquisition). A student may acquire knowledge from the course—passively, through listening and reading—while determining that active verbal engagement is unnecessary. This is in line with Bao (2020)‘s finding that not speaking up in class sometimes is a sign of quiet confidence because some students simply feel “confident enough to contemplate a less assertive disposition. (p. 9)” In addition, they may recognize the course’s value for knowledge acquisition, yet find it insufficient to justify the additional effort, risk, and anxiety involved in participating in class discussions. This is the exact instrumental, cost–benefit rationale that underpins our Strategic Disengagement Model.

Different from previous studies (e.g., Zhang et al., 2025), Self-Efficacy does not have a strong interaction effect. The lack of a strong correlation between Self-Efficacy and Silence Propensity can be explained in this way: It is a reasonable argument to suggest that students will be more likely to speak up assertively in class than to remain silent if they demonstrate a high level of English proficiency. However, as indicated in the quantitative data, there is no significant difference in perceived course value, and silence tendency among the different English proficiency groups, which means that no matter the students’ English proficiency, they all have low assessment of course value. That is, even students with high self-efficacy believe that the course is irrelevant in terms of enhancing their academic English proficiency within a four-week timeframe. One possible reason lies in the measurement of self-efficacy. Instead of measuring EAP-specific task confidence (such as confidence in comprehending a particular journal article), our scale measured general confidence. Students may feel generally competent yet find the course irrelevant to their immediate research needs. This measurement choice may have attenuated the link between self-efficacy and perceived course value. Additionally, quantitative results also indicated that Self-Efficacy does not moderate the relationship between Perceived Course Value and Silence Propensity. This means that even students with strong self-efficacy were inclined to remain silent when they perceived the course as lacking value. This finding reinforces the superiority of Perceived Course Value over individual learner dispositions in influencing strategic disengagement: The problem lies in students’ valuation of the effort required for speaking rather than their perceived ability to speak. But why do students experience this emotion? What external, context-driven factors contribute to the course’s low ratings, beyond the student-specific internal reasons identified in the interview findings?

While the interview data alone cannot fully answer these questions, several external factors merit consideration as plausible contextual influences. First, nowadays students do not rely exclusively on traditional classrooms; they engage in a concurrent, autonomous environment enriched by YouTube or Bilibili lessons, intensive exam-preparation videos, and AI-powered language models. These platforms provide rapid delivery, low redundancy, and the convenience of pause-and-resume learning. Therefore, the traditional classroom—characterized by a teacher-centered approach and lecture-based instruction—has significantly diminished its influence, especially in higher education. This issue is not confined to a single institution or cohort of learners; it is a widespread phenomenon in modern education (Awashreh, 2025). As a result, when students believe that knowledge can be obtained more effectively elsewhere, the perceived value of the classroom—and consequently, the EAP course—plummets. Second, the depreciation of English courses is further intensified by a social trend of diminishing instrumental interest in English studies, leading to the contraction of foreign language departments and a redefinition of English’s utility in public discourse (Zeng and Yang, 2024). These external shifts are not directly measured in the present study, but they offer a plausible macro-level context for understanding why students in our sample rated the course as low in value.

There is no easy victory against the tide of this era. The technological transformations, the diminishing instrumental usefulness of English, and the emergence of alternative learning ecosystems are structural forces that exceed the influence of any individual course or educator. Nonetheless, within the limited scope of this four-week EAP session, a small step forward remains possible—not via extensive pedagogical innovations, but through a conscious selection of reading materials for students. The solution may reside in selecting reading items that possess inherent intellectual appeal. When students are assigned generic textbook excerpts, they often dismiss the material as irrelevant to their academic goals, reducing reading to a mere obligatory task. However, when the emphasis is placed on research papers authored by masters and doctorate students from their own university in prestigious journals such as *Nature* and *Science*, the entire dynamic may change. Although this pedagogical approach was not empirically tested in this study, I speculate that it would reduce strategic disengagement by improving Perceived Course Value through academic identity and belonging—a mechanism that directly targets the central driver of our Strategic Disengagement Model. Put another way, such materials may increase students’ Perceived Course Value by indicating that the course is related to their own academic community, which would reduce the cost–benefit calculation that motivates strategic silence. Thus, what was previously regarded as an esoteric academic endeavor may transform into a lens through which people perceive a community they already belong to—a community where their capacity to contribute to avant-garde research appears possible. And reading will no longer be a compulsory obligation but a stimulus for curiosity, motivating individuals to dedicate time and effort to mastering the art of research.

Limitations of this study should be acknowledged to provide a clear direction for future research. First, interviewees were selected randomly rather than purposively based on their survey responses; a purposive sampling strategy might guarantee representation across various academic performance levels and English proficiency tiers (Creswell, 2014). Second, one methodological limitation concerns the reliability of the Reading Anxiety scale. Cronbach’s *α* for this scale was 0.64, which falls below the conventional threshold of 0.70. Several factors may explain this reduced reliability. First, the original scale was designed for general EFL reading scenarios, and when modified to fit an EAP learning environment, the underlying construct of reading anxiety may have manifested differently for this group of graduate students. Second, the four-week course duration did not provide sufficient opportunities for students to encounter a full range of anxiety-provoking reading experiences, which may have compressed the distribution of their responses and lowered the observed reliability. The third consideration is the scale’s brevity: as highlighted by Field (2018) and Taber (2018), short five-item measures regularly yield α coefficients in the 0.60 to 0.65 range. Even with this methodological precedent, I acknowledge that the scale would benefit from further refinement in future research. The third limitation concerns the exclusion of “face” as a measured construct. Although “face” concerns have been emphasized as crucial to comprehending silence in East Asian classrooms, our choice to leave them out was based on pilot observations of this specific adult graduate population, where “face”-related anxiety seemed less prominent than the perceived intellectual value of the discussion. However, because “face” was not directly measured, its explanatory power against perceived course value cannot be empirically compared. Future research that includes both constructs in the same design would help clarify their relative contributions.

## 6 Conclusion

This research examines the four-week EAP classroom as a distinct socio-academic microcosm, aiming to offer an alternative interpretation to the traditional diagnosis of “cultural shyness” and “anxiety” by foregrounding “rational disengagement” as a key mechanism resulting from a low assessment of the course. Quantitative results show that Perceived Course Value is the strongest predictor of Silence Propensity among the three independent variables. While this addresses the first research question, the qualitative data answers the second by uncovering multifaceted reasons (e.g., the academic research burden, the ease of AI technologies, the lack of necessity of reading and writing in English in some research fields, etc.) for the low ratings of the course. The integration of these etic and emic evidence yields a comprehensive depiction of EAP classroom silence among STEM graduate students in a research-intensive university context.

The importance of this study lies theoretically on that it questions the direct applicability of the “anxiety-face” model to all contexts. It calls for a situated understanding of silence, where course duration, perceived relevance, and assessment are important mediating variables. It elevates the concepts of instrumental motivation from peripheral to central explanatory positions in this specific EAP setting. Practically, this study offers evidence-based guidelines for the design of short-format EAP courses. The findings raise a broader question that goes beyond the immediate classroom context. As the status of English in higher education continues to shift due to the emergence of AI translation tools, and students’ access to alternative learning resources, college English instruction must adapt by clarifying its distinct value. Specifically, this study serves as a step toward turning what might otherwise remain a silent sprint into a collaborative dialogue. In doing so, it moves one step closer to the ambitious goal of reimagining language education in a rapidly changing academic landscape.

## Data Availability

The original contributions presented in the study are included in the article. Further inquiries can be directed to the corresponding author.

## References

[ref1] Ahmed A. (2024). A classroom ethnographic study on silence among EFL graduate students: a case study. Br. J. Transl. Linguist. Lit. 4, 02–17. doi: 10.54848/bjtll.v4i1.75

[ref2] Alhetah F. Alharbi M. A. (2026). Unheard or unwilling? Exploring the dynamics of silence and silencing in Saudi female EFL classrooms. Int. J. Appl. Linguist. doi: 10.1111/ijal.70097

[ref3] Awashreh R. (2025). Be aware: navigating challenges in AI-driven higher education. Int. J. Interdiscip. Educ. Stud. 20:203. doi: 10.18848/2327-011X/CGP/v20i01/203-218

[ref4] Bandura A. and National Inst of Mental Health (1986). Social Foundations of Thought and action: A Social Cognitive Theory. Englewood Cliffs, N.J: Prentice-Hall, Inc.

[ref5] Bao D. (2014). Understanding Silence and Reticence. Ways of Participating in second Language Acquisition. London: Bloomsbury Academic.

[ref6] Bao D. (2020). Exploring how silence communicates. Eng. Lang. Teach. Educ. J. 3, 1–13. doi: 10.12928/eltej.v3i1.1939

[ref7] Braun V. Clarke V. (2012). “Thematic analysis,” in APA Handbook of Research Methods in Psychology, Vol 2: Research Designs: Quantitative, Qualitative, Neuropsychological, and Biological, ed. H. Cooper (Washington, DC: American Psychological Association), 57–71. doi: 10.1037/13620-004

[ref8] Chemers M. M. Hu L. T. Garcia B. F. (2001). Academic self-efficacy and first year college student performance and adjustment. J. Educ. Psychol. 93, 55–64. doi: 10.1037/0022-0663.93.1.55

[ref9] Chen S. (2020). Classroom silence in college English class in China. US-China Foreign Lang. 18, 141–151. doi: 10.17265/1539-8080/2020.05.001

[ref10] Creswell J. W. (2014). Research Design: Qualitative, Quantitative, and Mixed Methods Approaches. 4th Edn. Thousand Oaks, CA: SAGE Publications.

[ref11] Dang V. T. (2024). English foreign language reading anxiety and reading strategies: a positive or negative correlation? SAGE Open 14, 1–13. doi: 10.1177/21582440241279663

[ref12] Deci E. L. Ryan R. M. (2012). “Motivation, personality, and development within embedded social contexts: an overview of self-determination theory,” in The Oxford Handbook of Human Motivation, ed. Ryan R. M. (New York, NY: Oxford University Press), 85–107.

[ref13] Eccles J. S. Wigfield A. (2002). Motivational beliefs, values and goals. Annu. Rev. Psychol. 53, 109–132. doi: 10.1146/annurev.psych.53.100901.135153, 11752481

[ref14] Fan H. (2023). Winter is coming? University teachers' and students' views on the value of learning English in China. Rev. Educ. 11, 1–18. doi: 10.1002/rev3.3410

[ref15] Field A. P. (2018). Discovering Statistics Using IBM SPSS Statistics. 5th Edn. Thousand Oaks, CA: SAGE Publications.

[ref16] Han M. (2016). A study on silence phenomenon in college English classroom. Int. J. Educ. Res. 4, 451–458.

[ref17] Hanh N. T. (2020). Silence is gold?: a study on students' silence in EFL classrooms. Int. J. High. Educ. 9, 153–160. doi: 10.5430/ijhe.v9n4p153

[ref18] Harumi S. (2024). Multi-contextual perspectives on silence: a narrative case study. Neofilolog 63, 265–292. doi: 10.14746/n.2024.63.2.3

[ref19] Hemade A. Malaeb D. Alhuwailah A. . (2025). The 9-item academic self-efficacy (ASE) scale: validity, reliability and measurement invariance across sexes and six Arab nations. Sci. Rep. 15:12620. doi: 10.1038/s41598-025-96896-6, 40221563 PMC11993676

[ref20] King J. Harumi S. (Eds.). (2020). East Asian Perspectives on Silence in English Language Education, (vol. 6) Bristol, UK: Multilingual Matters.

[ref21] Langen I. Stamov Roßnagel C. (2023). East is east: Socratic classroom communication is linked to higher stress in students from Confucian heritage cultures. Heliyon 9:e15748. doi: 10.1016/j.heliyon.2023.e15748, 37251875 PMC10209327

[ref22] Li H. (2022). Classroom enjoyment: relations with EFL students' disengagement and burnout. Front. Psychol. 12:824443. doi: 10.3389/fpsyg.2021.824443, 35095702 PMC8792741

[ref23] Liu M. (2005). Reticence in oral English language classrooms: a case study in China. TESL Rep. 38, 1–16.

[ref24] Liu M. (2006). Reticence in oral English classrooms: causes and consequences. Asian J. Engl. Lang. Teach. 16, 45–66. doi: 10.65961/ajelt-2006-1-003

[ref25] Liu Y. Liu M. Zhang S. (2026). Interpersonal satisfaction and classroom silence in Chinese universities: the mediating role of social anxiety. Front. Psychol. 17:1676791. doi: 10.3389/fpsyg.2026.1676791, 41640883 PMC12864520

[ref26] Maher K. King J. (2022). ‘The silence kills me.’: ‘Silence’ as a trigger of speaking-related anxiety in the English-medium classroom. Engl. Teach. Learn. 46, 213–234. doi: 10.1007/s42321-022-00119-4

[ref27] Maher K. King J. (2023). Language anxiety and learner silence in the classroom from a cognitive-behavioral perspective. Annu. Rev. Appl. Linguist. 43, 105–111. doi: 10.1017/S0267190523000077

[ref29] Peng Y. (2024). Balancing silence and participation: enhancing classroom engagement among Chinese EFL students. J. Silenc. Stud. Educ. 4, 24–38. doi: 10.31763/jsse.v

[ref30] Pham C. K. Chong S. L. Wan R. (2023). A phenomenographic research study of students’ conceptions of silence in face-to-face English as a foreign language learning. SAGE Open 13, 1–14. doi: 10.1177/21582440231216343

[ref31] Shachter J. Haswell C. G. (2022). Exploring ways of accommodating silent Japanese language learners in the classroom: insights from scholars in the field. J. Silenc. Stud. Educ. 1, 70–81. doi: 10.31763/jsse.v1i2.12

[ref32] Sheeran P. Webb T. L. (2016). The intention–behavior gap. Soc. Personal. Psychol. Compass 10, 503–518. doi: 10.1111/spc3.12265

[ref34] Su Y. Chu X. (2024). Study on the causes and effects of foreign language learning anxiety among Chinese college students. Trans. Soc. Sci. Educ. Humanit. Res. 6, 63–70. doi: 10.62051/0p9tra44

[ref35] Taber K. S. (2018). The use of Cronbach's alpha when developing and reporting research instruments in science education. Res. Sci. Educ. 48, 1273–1296. doi: 10.1007/s11165-016-9602-2

[ref36] The Economist. (2024). “Why China is losing interest in English?”

[ref37] Van Zyl L. E. Klibert J. Shankland R. See-To E. W. K. Rothmann S. (2022). The general academic self-efficacy scale: psychometric properties, longitudinal invariance, and criterion validity. J. Psychoeduc. Assess. 40, 777–789. doi: 10.1177/07342829221097174

[ref38] Wang M. (2019). Analysis of classroom silence in English class in Chinese universities. Acad. J. Humanit. Soc. Sci. 2, 54–64. doi: 10.25236/AJHSS.040024

[ref39] Wei W. Cao Y. (2024). Willing, silent or forced participation? Insights from English for academic purposes classrooms. RELC J. 55, 63–78. doi: 10.1177/00336882211066619

[ref40] West T. (2025). Qualitative Study of Faculty Perceptions of Student Success in Online 16-Week Versus Accelerated Courses at Community Colleges in Houston, Texas. Doctoral dissertation, Lamar University-Beaumont.

[ref41] Yang L. Lin Y. (2024). To talk or to remain silent? Foreign language enjoyment and perceived classroom climate as drivers of change in Chinese EFL learners’ willingness to communicate: a latent growth curve analysis. Innov. Lang. Learn. Teach. 1–18, 1–18. doi: 10.1080/17501229.2024.2391376, 37339054

[ref42] Yuan L. (2021). ‘Reversing gears’: China increasingly rejects English, and the world. The New York Times.

[ref43] Yue Y. Jia Y. Wang X. (2022). Self-efficacy and negative silence in the classroom: the mediating role of fear of negative evaluation. Nurse Educ. Pract. 62:103379. doi: 10.1016/j.nepr.2022.103379, 35738157

[ref44] Zeng J. Yang J. (2024). English language hegemony: retrospect and prospect. Hum. Soc. Sci. Commun. 11, 1–9. doi: 10.1057/s41599-024-02821-z

[ref45] Zhang J. Fang Z. Rhee G. S. (2025). Analysis of classroom silence behaviors among Chinese and Korean undergraduates. Front. Psychol. 16:1674145. doi: 10.3389/fpsyg.2025.1674145, 41426400 PMC12713118

[ref46] Zhao J. (2016). The reform of the National Matriculation English Test and its impact on the future of English in China: will English lose its predominance in the Chinese foreign language landscape? Engl. Today 32, 38–44. doi: 10.1017/S0266078415000681

[ref47] Zhao M. (2018). Educational investigation and cultural origins of classroom silence. Teach. Admin. 27, 87–89.

[ref48] Zhu H. O’Sullivan H. (2022). Shhhh! Chinese students are studying quietly in the UK. Innov. Educ. Teach. Int. 59, 275–284. doi: 10.1080/14703297.2020.1813603

[ref49] Zhuang P. (2023). Investigating the origins of silence in college English classrooms and exploring potential remedies. Front. Educ. Res. 6, 42–47. doi: 10.25236/FER.2023.061708

